# Early Detection of Age-Associated Cellular Decline: Report of an Expert Consensus

**DOI:** 10.1093/geroni/igaa057.2759

**Published:** 2020-12-16

**Authors:** Jack Guralnik, Matteo Cesari, Ariel Beresniak, Leocadio Rodriguez-Manas, Antonio Cherubini

**Affiliations:** 1 University of Maryland School of Medicine, Baltimore, Maryland, United States; 2 University of Milan, Milan, Lombardia, Italy; 3 Data Mining International, Geneva, Geneve, Switzerland; 4 University Hospital of Getafe, Getafe, Madrid, Spain; 5 INRCA, Ancona, Marche, Italy

## Abstract

Cellular processes often decline with age and cells lose their ability to function optimally, which may lead to organ-specific dysfunction and the development of systemic age-related diseases. The cellular hallmarks of aging are associated with clinical signs and symptoms and can be termed Age Associated Cellular Decline. An expert consensus study group was convened to provide an initial framework for the development of a tool for adults over 50 years old, which identifies self-reported symptoms and observable signs likely to be early and/or surrogate markers of age associated cellular decline. A total of 16 potential early signs and symptoms of age associated cellular decline were identified and need to be validated in further research.

